# Cryptorchidism: Effects of Maternal Diabetes or PBDEs

**DOI:** 10.1289/ehp.11096

**Published:** 2008-05

**Authors:** Gavin W. ten Tusscher, Janna G. Koppe

**Affiliations:** Department of Pediatrics and Neonatology, Westfriesgasthuis, Hoorn, the Netherlands; ECOBABY Foundation, Loernersloot, the Netherlands, E-mail: Janna.Koppe@inter.nl.net

It was with great interest that we read the article by Main et al. (2007) regarding polybrominated diphenyl ethers (PBDEs) and cryptochordism, and we are impressed with the data in humans. Main et al. stated that the concentration of PBDEs in breast milk was significantly higher in boys with cryptorchidism compared with controls. It is certainly possible that there is a link between fetal PBDE exposure and cryptorchidism; however, we noted that the cohort included children of diabetic mothers. Of the 33 boys with cryptorchidism, 4 in the Finnish group and 1 of 28 in the Danish breast milk–sample group had diabetic mothers. It is widely known that diabetes is a major cause of congenital malformations, and these malformations are dependent on the severity of the diabetes. Therefore, you cannot simply match by diabetes between cases and controls. In a study of 173 mothers with diabetes, we found that 10% of the offspring had congenital malformations related to the severity of the diabetes, classified according to the Priscilla White classification (Koppe et al. 1983). Virtanen et al. (2006), together with Main, published a study reporting an increased risk of cryptorchidism following mild gestational diabetes. In our opinion, the cases of mothers with diabetes should be excluded from analysis of congenital malformations, both in the breast milk group and the placenta group reported by Main et al. (2007).

The group of mothers with diabetes is in itself an interesting group. Are the placenta and breast milk levels of PBDEs or the fat content different between the diabetic cases and the others?

In general, because most PBDEs have phenobarbital-like effects, it seems plausible that they should cause an increase in congenital malformations, such as is seen with phenobarbital (Dessens et al. 1994; Koppe et al. 1973).
